# Multiple Functions of Draxin/Netrin-1 Signaling in the Development of Neural Circuits in the Spinal Cord and the Brain

**DOI:** 10.3389/fnana.2021.766911

**Published:** 2021-11-25

**Authors:** Giasuddin Ahmed, Yohei Shinmyo

**Affiliations:** ^1^Department of Neuroscience and Pharmacology, The University of Texas Southwestern Medical Center, Dallas, TX, United States; ^2^Department of Medical Neuroscience, Graduate School of Medical Sciences, Kanazawa University, Kanazawa, Japan

**Keywords:** axon guidance, draxin, netrin-1, spinal commissural axons, corpus callosum, thalamocortical projections, glycoproteins

## Abstract

Axon guidance proteins play key roles in the formation of neural circuits during development. We previously identified an axon guidance cue, named draxin, that has no homology with other axon guidance proteins. Draxin is essential for the development of various neural circuits including the spinal cord commissure, corpus callosum, and thalamocortical projections. Draxin has been shown to not only control axon guidance through netrin-1 receptors, deleted in colorectal cancer (Dcc), and neogenin (Neo1) but also modulate netrin-1-mediated axon guidance and fasciculation. In this review, we summarize the multifaceted functions of draxin and netrin-1 signaling in neural circuit formation in the central nervous system. Furthermore, because recent studies suggest that the distributions and functions of axon guidance cues are highly regulated by glycoproteins such as Dystroglycan and Heparan sulfate proteoglycans, we discuss a possible function of glycoproteins in draxin/netrin-1-mediated axon guidance.

## Introduction

The proper function of the nervous system relies on appropriate patterns of connectivity among an enormous number of neurons. A key process in the formation of neural circuits is the navigation of neuronal axons to their targets, which is regulated by axon guidance molecules (Tessier-Lavigne and Goodman, 1996; Dickson, 2002). In the 1990s, four major families of axon guidance proteins were identified: netrins, semaphorins, ephrins, and slits (Raper and Kapfhammer, 1990; Rothberg et al., 1990; Kolodkin et al., 1993; Luo et al., 1993; Kennedy et al., 1994; Serafini et al., 1994; Cheng et al., 1995; Drescher et al., 1995; Brose et al., 1999; Kidd et al., 1999). In addition, three other families of secreted signaling molecules have been shown to act as axon guidance cues: the Wingless/Wnt, Hedgehog (Hh), and transforming growth factor-β/bone morphogenic protein (BMP) families (Charron and Tessier-Lavigne, 2007; Zou and Lyuksyutova, 2007; Yam and Charron, 2013). Numerous studies of these axon guidance cues have revealed the molecular basis of neural circuit formation, in which axons expressing guidance receptors navigate to their targets by detecting a variety of attractive and repulsive cues presented by cells in the environment. However, many guidance processes during brain development cannot be explained by the function of these molecules alone. It has been shown that interactions of multiple guidance signals diversify the possible responses to a limited number of guidance cues (Dudanova and Klein, 2013). Another potential mechanism is that more unidentified molecules controlling axon guidance and/or regulating known guidance signaling participate in the formation of the complex nervous system.

We previously identified a novel axon guidance cue, draxin, that is highly expressed in the developing central nervous system (CNS) (Islam et al., 2009). We showed that draxin controls axon guidance through netrin-1 receptors, Deleted in colorectal cancer (Dcc) and Neogenin (Neo1) (Ahmed et al., 2011; Shinmyo et al., 2015). Draxin has also been shown to modulate netrin-1-mediated axon guidance and fasciculation (Gao et al., 2015; Liu et al., 2018; Meijers et al., 2020). In this review, we highlight possible roles of draxin/netrin-1 signaling in the development of spinal commissural axons, corpus callosum, and thalamocortical axons. Furthermore, we discuss a possible contribution of glycoproteins to the distribution and function of draxin and netrin-1 in the developing spinal cord and brain.

## Draxin and Netrin-1 Signaling in Axon Guidance

Draxin is a secreted protein that has a cysteine-rich domain in its carboxyl terminal region. Although the positions of 10 cysteine residues in the cysteine-rich domain of draxin are similar to those of members of the Dickkopf (Dkk) family, draxin shares no homology with any known proteins (Islam et al., 2009; Miyake et al., 2009). Draxin homologs are found in vertebrate genomes but not in invertebrate genomes. We and others reported that draxin regulates the outgrowth of axons originating from various types of neurons *in vitro* (Islam et al., 2009; Naser et al., 2009; Ahmed et al., 2010, 2011; Chen et al., 2013; Meli et al., 2015; Shinmyo et al., 2015). In addition, *draxin* knockout (KO) mice show defects in many axonal tracts including the spinal cord commissure, anterior commissure, corpus callosum, internal capsule (thalamocortical and corticofugal tracts), and hippocampal commissure (Islam et al., 2009; Shinmyo et al., 2015). Thus, draxin controls the development of neural circuits in the CNS via a vertebrate-specific mechanism.

**FIGURE 1:**
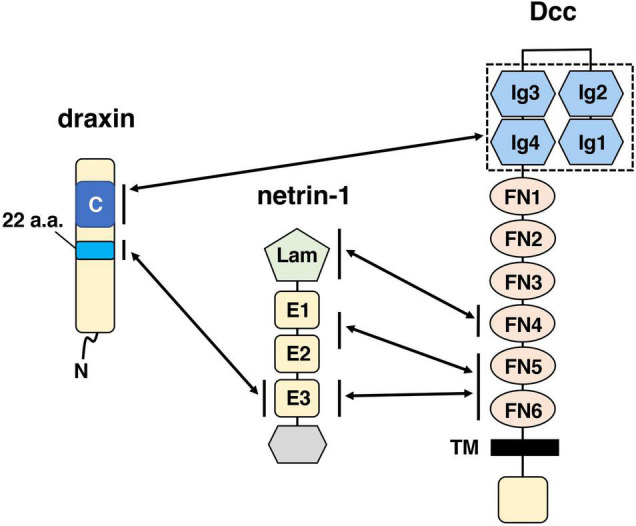
Schematic drawings of draxin and its interacting proteins, netrin-1, and Dcc. The C-terminal domain of draxin interacts with the Ig domain of Dcc. A 22-residue sequence of draxin interacts with the EGF-3 (E3) domain of netrin-1. Netrin-1 interacts with Dcc at the fibronectin (FN) domain through three binding sites: the laminin VI domain (Lam), EGF-1/EGF-2 (E1/E2) domain, and E3 domain. It should be noted that in addition to Dcc, Neo1 is also a functionally important receptor for draxin. Because Dcc and Neo1 are structurally very similar proteins, the C-terminal domain of draxin may interact with the Ig domain of Neo1.

The neuronal network defects in *draxin* KO mice clearly demonstrate that draxin plays an essential role in axon guidance in the spinal cord and the brain. Yet, how draxin regulates axon guidance *in vivo* is still poorly understood. Through cell binding assays, we previously showed that draxin binds the netrin-1 receptors Dcc, Neo1, Unc5s, and Down’s syndrome cell adhesion molecule (DSCAM) (Ahmed et al., 2011). Based on *draxin* and *Dcc* KO mouse phenotype similarities, we analyzed interaction between draxin and Dcc further and found that draxin co-immunoprecipitated immunoglobulin (Ig) domain of Dcc, which is separate from the netrin-1-binding domain (Figure 1). Furthermore, draxin-mediated axonal outgrowth in neurons from the neocortex (Ahmed et al., 2011; Meli et al., 2015; Shinmyo et al., 2015), thalamus (Shinmyo et al., 2015), and dorsal horn of the spinal cord (Chen et al., 2013) requires Dcc and Neo1. A recent study showed draxin directly binds netrin-1 and acts as a netrin-1 antagonist by preventing netrin-1 from binding to the Dcc receptor (Gao et al., 2015). On the other hand, structural studies demonstrated that draxin can interact with both netrin-1 and Dcc simultaneously (Liu et al., 2018). Draxin contains the 22-residue conserved hydrophobic sequence that binds netrin-1 at the EGF-3 domain and an approximately 90-residue cysteine knot domain at the C terminus that binds Dcc at the Ig4 domain (Figure 1). Netrin-1 utilizes its laminin VI, EGF-1, EGF-2, and EGF-3 domains to interact with the fibronectin (FN) domain of Dcc (Figure 1; Finci et al., 2014; Xu et al., 2014). From the binding configurations of these three molecules, Liu et al. (2018) have proposed that the draxin/netrin-1 complex can build a bridge between two axons decorated with Dcc, promoting adhesion, and fasciculation between these axons. Thus, it is likely that draxin not only controls axon guidance through the Dcc/Neo1 receptors but also modulates netrin-1-mediated axon guidance and fasciculation. In contrast, it is unknown whether Unc5s and DSCAM mediate draxin functions in axon guidance and fasciculation.

## Spinal Commissural Axons

**FIGURE 2:**
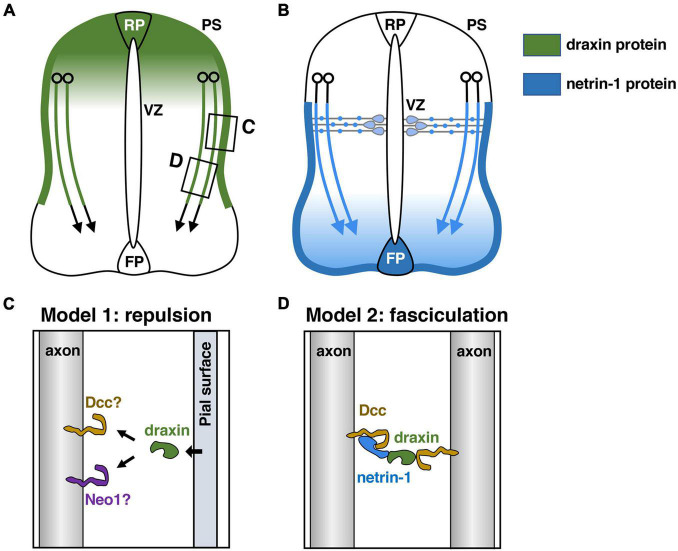
Draxin and netrin-1 signaling in the guidance of spinal commissural axons. **(A)** *Draxin* mRNA is expressed in the dorsal spinal cord including the roof plate (RP), and its protein is deposited along the pial surface of the dorsal spinal cord and commissural axons. VZ, ventricular zone; PS, pial surface. **(B)** Netrin-1 produced by progenitor cells in the ventricular zone and the floor plate (FP) cells acts synergistically to guide commissural axons in the spinal cord. Note that netrin-1 is deposited along the pial surface of the ventral spinal cord and commissural axons. **(C,D)** Two models for draxin functions in the guidance of spinal commissural axons. The areas within the boxes in panel **(A)** were magnified and are shown in panels **(C,D)**. We have proposed that draxin on the pial surface acts as a repulsive barrier for commissural axons **(C)**. Liu et al. (2018) have proposed a model in which draxin mediates the fasciculation of Dcc-expressing axons by creating a Dcc-draxin-netrin-1-Dcc bridge-like configuration between two axons **(D)**.

Commissural axon guidance has been extensively studied in the developing spinal cord (Tessier-Lavigne and Goodman, 1996; Dickson, 2002; Kolodkin and Tessier-Lavigne, 2011). Commissural neurons are generated in the dorsal spinal cord and extend their axons ventrally toward the floor plate (FP), turning away from the roof plate (RP) and lying close to the pial surface (Figure 2A). Once they cross the ventral midline, most of the commissural axons turn rostrally toward the brain.

The RP is the source of diffusible repellents that guide the early trajectory of spinal commissural axons (Augsburger et al., 1999). It was shown that Growth/differentiation factor 7 (Gdf7): Bmp7 heterodimers mediate the RP-derived chemorepellent activity for spinal commissural axons (Augsburger et al., 1999; Butler and Dodd, 2003). Indeed, commissural axons extend aberrantly toward or into the RP in *Bmp7* and *Gdf7* mutant mice (Butler and Dodd, 2003). Since many commissural axons in *Bmp7* and *Gdf7* doubly mutant mice recover their correct projection pattern at later developmental stages, it has also been postulated that the RP may express other repellents (Butler and Dodd, 2003). *Draxin* mRNA is expressed in the dorsal spinal cord including the RP (Islam et al., 2009). *In vivo* experiments with chicken spinal cord as well as *in vitro* neurite outgrowth experiments showed that draxin has repulsive activity for spinal commissural axons (Islam et al., 2009). Thus, draxin is most likely an additional repellent protein from the dorsal spinal cord that orients commissural axons (Figure 2A). It should be noted that *draxin* KO mice do not show medial misrouting of commissural axons (Islam et al., 2009). This may reflect functional redundancy with Bmp7 and Gdf7 expressed by the RP. Further analyses with double or triple mutant mice for these factors would help to clarify this point.

In contrast to the RP, the FP was shown by *in vitro* explant assays to be the source of chemoattractants for commissural axons (Tessier-Lavigne et al., 1988; Placzek et al., 1990). So far, three FP-derived chemoattractants have been identified: netrin-1 (Kennedy et al., 1994; Serafini et al., 1994), sonic hedgehog (Shh) (Charron et al., 2003), and vascular endothelial growth factor (VEGF) (Ruiz de Almodovar et al., 2011). In the mouse spinal cord, *netrin-1* is expressed in not only FP cells but also neural progenitor cells (NPCs) of the ventricular zone (VZ) (Serafini et al., 1996). Recently, conditional knockout approaches have been used to investigate the roles of netrin-1 derived from these two sources in commissural axon guidance. Removal of netrin-1 from the FP results in the defasciculation and misrouting of commissural axons in the ventral spinal cord, supporting the classical model that FP-derived netrin-1 guides commissural axons at long range (Figure 2B; Moreno-Bravo et al., 2019; Wu et al., 2019). Furthermore, removal of both FP-netrin-1 and the Shh receptor Boc results in additive guidance defects, suggesting that netrin-1 from the FP collaborates with Shh in commissural axon guidance in the ventral spinal cord (Wu et al., 2019). Interestingly, draxin and netrin-1 proteins form reciprocal gradients along the dorsoventral axis of the spinal cord. Gao et al. (2015) have proposed that draxin may sharpen the extracellular distribution of active netrin-1 by acting as a netrin-1 antagonist.

Although removal of netrin-1 from the FP causes the axon guidance defects mentioned above, many commissural axons reach the midline in the spinal cord. This is in contrast to the phenotype in *netrin-1* hypomorphs (Serafini et al., 1996) and *netrin-1*-null mutants (Bin et al., 2015; Yung et al., 2015), in which the number of commissural axons reaching the FP is substantially reduced. Interestingly, several recent studies demonstrated that netrin-1 from the VZ, rather than the FP, is the key source of netrin-1 that guides commissural axons to the FP in the spinal cord and the hindbrain (Dominici et al., 2017; Varadarajan et al., 2017; Yamauchi et al., 2017). In the spinal cord, removal of netrin-1 from the VZ results in many guidance defects of commissural axons, which aberrantly cross the dorsal midline and invade the VZ and motor neuron domain (Dominici et al., 2017; Varadarajan et al., 2017). This result suggests that VZ-supplied netrin-1 is crucial for commissural axon guidance to the midline (Figure 2B).

How does VZ-derived netrin-1 guide commissural axons to the ventral midline? While *netrin-1* mRNA is expressed in NPCs of the VZ, its protein is present on the pial surface and commissural axons (Figure 2B; Kennedy et al., 2006; Varadarajan et al., 2017). It was suggested that netrin-1 produced by NPCs is transported via their radial processes to the basement membrane, where their end-feet contact the pial surface (Varadarajan et al., 2017). Thus, netrin-1 deposited on the pial surface is likely to function as a short-range cue for commissural axons, which grow alongside it (Figure 2B). One current model suggests that the pial-netrin-1 substrate acts by haptotaxis to promote commissural axon extension and direct axons toward the ventral midline. Other excellent papers detailed the functions of netrin-1 in guiding the commissural axons to the ventral midline (Morales, 2018; Chedotal, 2019; Comer et al., 2019; Ducuing et al., 2019). Importantly, draxin is also heavily deposited in the basement membrane along the dorsal and lateral sides of the spinal cord (Figure 2A; Islam et al., 2009), suggesting a role of pial-draxin in commissural axon guidance. In *draxin* KO mice, commissural axons project in a defasciculated manner toward the FP (Islam et al., 2009). In addition, commissural axons along the basement membrane are observed more frequently in *draxin* KO mice than in control mice (Islam et al., 2009). Based on these results, we have proposed that draxin on the pial surface acts as a repulsive barrier for commissural axons (Figure 2C). In this context, it will be interesting to determine how Dcc and/or other receptors mediate signaling cascades for draxin and netrin-1 from the pial surface, especially because these two ligands have opposite activities on spinal cord commissural axons. Another potential explanation for this phenotype is that draxin promotes the fasciculation of commissural axons by interacting with Dcc and netrin-1 (Liu et al., 2018; Meijers et al., 2020). Liu et al. (2018) have proposed a model in which draxin mediates the fasciculation of Dcc-expressing axons by creating a Dcc-draxin-netrin-1-Dcc bridge-like configuration between two axons (Figure 2D; Liu et al., 2018; Meijers et al., 2020). This model is supported by observations that these three proteins are present on commissural axons and that the accumulation of netrin-1 protein is greatly reduced in axons of *Dcc* mutants (Varadarajan et al., 2017).

It is important to note that draxin also interacts with the canonical Wnt receptor LRP6 and antagonizes canonical Wnt signaling (Miyake et al., 2009). It was shown that canonical Wnt signaling is required for postcrossing commissural axon guidance in the spinal cord (Aviles and Stoeckli, 2016). Although postcrossing commissural axon projections seemed to be not affected in *draxin* KO mice (Islam et al., 2009), draxin might be involved in postcrossing commissural axon guidance by modulating canonical Wnt signaling.

## The Corpus Callosum

The corpus callosum is the major axonal tract that connects and coordinates information between the two cerebral hemispheres. Complete absence (agenesis) of the corpus callosum in humans is associated with various neurological disorders such as language dysfunction and abnormalities in social interaction (Paul et al., 2007; Edwards et al., 2014; Brown and Paul, 2019). A large number of genes including *netrin-1*, *draxin* and *Dcc* have been shown to be linked to malformations of the corpus callosum in humans and mice (Richards et al., 2004; Paul et al., 2007; Edwards et al., 2014). Loss-of-function mutations of *DCC* in humans cause dysgenesis of the corpus callosum (Morcom et al., 2021b). Although pathogenic variants in *DRAXIN* and *NETRIN-1* have not yet been reported in humans with dysgenesis of the corpus callosum, in mice, mutant forms of *draxin, netrin-1*, and *Dcc* have each been shown to cause agenesis of the corpus callosum with similar misprojection of callosal axons (Serafini et al., 1996; Fazeli et al., 1997; Ren et al., 2007; Islam et al., 2009; Fothergill et al., 2014). In these mutants, callosal axons do not cross the midline; instead, they form ipsilateral “Probst” bundles that run parallel to the midline. These facts suggest that these three molecules may act in a similar manner to control midline crossing of callosal axons.

During brain development, callosal pioneering axons originate in the cingulate cortex, and cross the midline in a region initially separated by the interhemispheric fissure (IHF) (Rakic and Yakovlev, 1968; Silver et al., 1982; Gobius et al., 2016). Subsequently, these pioneering axons guide the midline crossing of neocortical callosal axons projecting from mainly layers II/III and V. Two distinct developmental mechanisms have been shown to be critical for midline crossing of callosal axons. First, the remodeling of the IHF mediated by specialized astroglial cells known as midline zipper glia (MZG) is necessary to form a permissive substrate for callosal pioneering axons to cross the midline (Gobius et al., 2016). Second, midline glial structures that express axon guidance cues including netrin-1, draxin, and Slit2 play an essential role in the guidance of callosal axons at the midline (Lindwall et al., 2007; Nishikimi et al., 2013; Fothergill et al., 2014). Recently, Morcom et al. (2021a; 2021b) reported that draxin, netrin-1 and Dcc are key players in the development of astroglial cells during IHF remodeling. It is therefore plausible that draxin/netrin-1 signaling has multiple roles in corpus callosum formation including IHF remodeling and the guidance of callosal axons.

**FIGURE 3:**
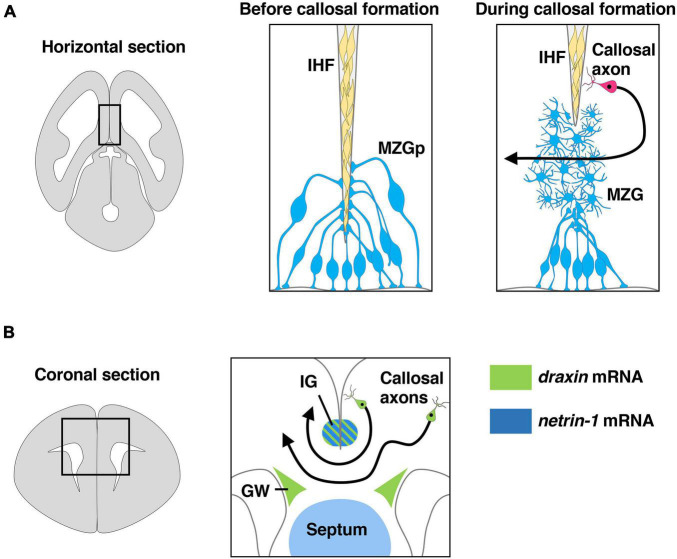
Draxin and netrin-1 signaling in the development of corpus callosum. **(A)** The remodeling of the interhemispheric fissure (IHF) mediated by midline zipper glia (MZG) is necessary to form a permissive substrate for callosal axons to cross the midline. The area within the box in the left panel is shown in the middle panel and the right panel. Draxin, netrin-1, and Dcc are key players in IHF remodeling. MZGp, midline zipper glia progenitors. **(B)** Midline structures that express draxin and netrin-1 paly an essential role in guidance of callosal axons at the midline. The area within the box in the left panel is shown in the right panel. *Draxin* mRNA is expressed in the indusium griseum (IG) and glial wedge (GW), while *netrin-1* mRNA is expressed in IG and septum. *Draxin* mRNA is also expressed in callosal neurons.

Interhemispheric fissure remodeling has been shown to consist of several key processes including generation and specification of the MZG from radial glia, somal translocation of the MZG to the IHF and differentiation of the MZG into multipolar MZG cells that degrade the IHF and intercalate across the midline (Figure 3A; Gobius et al., 2016; Morcom et al., 2021a,b). A previous study with neuroimaging showed that agenesis of the corpus callosum is remarkably correlated with retention of the IHF in humans (Gobius et al., 2016). Furthermore, a more recent study showed that IHF remodeling is disrupted in humans with *DCC* pathogenic variants that display agenesis of the corpus callosum (Morcom et al., 2021b). Thus, developmental defects in IHF remodeling are likely to be a primary etiology leading to human callosal agenesis (Gobius et al., 2016). Consistent with human subjects with *DCC* mutations, both *netrin-1* and *Dcc* mutant mice display impaired remodeling of the IHF (Hakanen and Salminen, 2015; Morcom et al., 2021b). *Dcc* mutant mice show irregular morphology of the MZG and reduced somal translocation to the IHF. Taken together with the fact that *netrin-1* and *Dcc* are expressed in the MZG during IHF remodeling, these results suggest that netrin-1/Dcc signaling facilitates IHF remodeling by regulating MZG morphology and migration to the IHF (Morcom et al., 2021b).

It remains unclear whether IHF remodeling is disrupted in *draxin* KO mice. Recently, however, an eight base-pair deletion in the *draxin* gene was identified in BTBR mouse strains, which have a severe commissural phenotype including dysgenesis of the corpus callosum (Morcom et al., 2021a). This deletion results in a premature strop codon in exon 2 of the *draxin* gene, creating a truncated protein lacking domains that bind netrin-1 and Dcc. Importantly, the severity of corpus callosum dysgenesis in BTBR mice was shown to be strongly associated with abnormal retention of the IHF, suggesting that a failure of IHF remodeling caused by loss of draxin function results in corpus callosum dysgenesis in these mice. *Draxin* is highly expressed in MZG progenitors, and its protein is widely distributed on various components of the interhemispheric midline including the MZG, leptomeninges, and callosal axons. Interestingly, BTBR mice show increased somal translocation of the MZG to the IHF, which is the opposite phenotype of *Dcc* mutant mice. In this context, draxin might antagonize netrin-1/Dcc signaling that stimulates somal translocation of the MZG to the IHF during normal IHF remodeling (Morcom et al., 2021a). Furthermore, BTBR mice show an increase in the proliferation of both MZG cells and leptomeningeal cells. This phenotype is not observed in *Dcc* and *netrin-1* mutants, suggesting that draxin may regulate the proliferation of these cells independently of netrin-1/Dcc signaling (Morcom et al., 2021a). Thus, draxin may be involved in multiple processes of IHF remodeling by interacting with not only netrin/Dcc signaling but also other signaling molecules (Morcom et al., 2021a).

Midline crossing of callosal axons is thought to be controlled by axon guidance cues expressed in midline guidepost structures, including the glial wedge (GW) and indusium griseum (IG) (Figure 3B; Lindwall et al., 2007; Nishikimi et al., 2013; Fothergill et al., 2014). *Netrin-1* is expressed in the septum and the IG region (Figure 3B), and *Dcc* is expressed by callosal axons during midline crossing of callosal axons (Fothergill et al., 2014; Hakanen and Salminen, 2015). *In vitro* experiments showed that netrin-1 acts as a chemoattractant for callosal pioneering axons derived from the cingulate cortex but is not attractive for neocortical callosal axons, suggesting that netrin-1 attracts callosal pioneering axons toward the midline (Fothergill et al., 2014). Furthermore, netrin-1/Dcc signaling was suggested to attenuate Slit2-mediated repulsion of pre-crossing axons from the GW and the IG, allowing them to approach and cross the midline (Fothergill et al., 2014). During midline crossing of callosal axons, they must decrease responsiveness to attractive cues from midline guidepost structures and increase responsiveness to repulsive cues from these structures to leave the midline and enter the contralateral hemisphere. Importantly, Dcc expression is downregulated on post-crossing callosal axons. This leads to the cancelation of netrin-1-regulated attenuation of Slit2-mediated repulsion, thereby allowing the callosal axons to leave the midline (Fothergill et al., 2014).

We previously showed that draxin regulates axonal growth of neocortical neurons in a concentration-dependent manner and that this effect is mediated through the Dcc and Neo1 receptors (Shinmyo et al., 2015). Low concentrations of draxin stimulate neurite outgrowth of neocortical neurons, while higher concentrations of draxin inhibit neurite outgrowth. This result suggests that the bimodal effects of draxin might be critical for midline crossing of callosal axons. Similar bimodal responses depending on concentration were reported for ephrin-A2 (Hansen et al., 2004) and Shh (Kolpak et al., 2005) on retinal ganglion cells. Ephrin-A2 inhibits retinal axon outgrowth at high concentrations but promotes growth at lower concentrations. This concentration-dependent activity of ephrin-A2 has been proposed to be involved in topographic map formation in the mouse visual system (Hansen et al., 2004). During corpus callosum development, *draxin* is expressed in midline glial structures including the GW and the IG (Figure 3B). It may be possible that draxin from the midline glial structures initially attracts callosal axons toward the midline and later acts as a repellent when callosal axons are exposed to higher concentrations of draxin at the midline. Taken together, the current evidence suggests that draxin from the midline structures is likely to serve critical roles in the development of the corpus callosum through the regulation of IHF remodeling and the guidance of callosal axons. However, it is also possible that draxin secreted from other regions contributes to the guidance of callosal axons. It should be noted that draxin is also expressed in callosal neurons of the cingulate cortex and the neocortex (Figure 3B), suggesting that draxin might regulate midline crossing of callosal axons in an autocrine manner. Conditional strategies that enable the spatial and temporal manipulation of draxin expression are necessary to determine the exact roles of draxin in the development of the corpus callosum.

## Thalamocortical Projections

**FIGURE 4:**
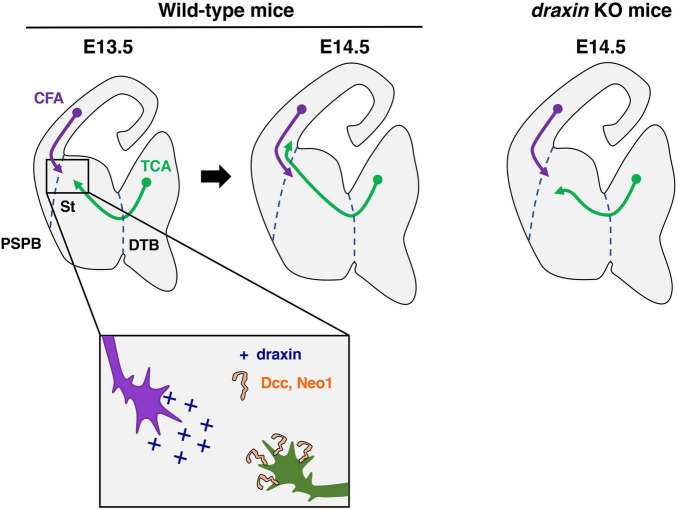
Draxin signaling in the development of thalamocortical axons. Thalamocortical axons (TCA) and corticofugal axons (CFA) meet in the striatum (St) to form the internal capsule around E13.5. At E14.5, and TCA progress through the pallial-subpallial boundary (PSPB) to enter the neocortex in wild-type mice. In *draxin* KO mice, TCA do not cross the PSPB, while early CFA projected normally toward the internal capsule. We have proposed a model, in which draxin from CFA promotes the growth of TCA from the internal capsule to the neocortex, and this effect of draxin is mediated through the Dcc and Neo1 receptors. DTB, diencephalic/telencephalic boundary.

Thalamocortical axons originate in the dorsal thalamus and carry sensory information to the neocortex (Lopez-Bendito and Molnar, 2003; Leyva-Diaz and Lopez-Bendito, 2013; Garel and Lopez-Bendito, 2014). In mice, developing thalamocortical axons first exit the thalamus and then turn dorsolaterally at the diencephalic–telencephalic boundary (DTB) to enter the internal capsule by embryonic day 13 (E13) (Figure 4). Thalamic axons continue to extend through the subpallium and reach the pallial-subpallial boundary (PSPB), where they meet corticofugal axons, at around E14 (Figure 4). Thalamic axons advance through the intermediate zone to reach the developing cortex and arrive at the appropriate cortical regions around E16. Thalamic axons wait in the subplate for 2 or 3 days and finally invade the cortical plate, forming synapses onto neurons of the appropriate layers.

It was shown over two decades that the guidance of thalamocortical axons is dependent on neocortical subplate neurons, which pioneer the corticofugal pathway from the neocortex to the internal capsule (McConnell et al., 1989; Ghosh et al., 1990; Ghosh and Shatz, 1993). The handshake hypothesis, proposed by Molnar and colleagues, postulates that thalamocortical projections rely on the interaction of corticofugal and thalamocortical axons (Molnar et al., 1998). This hypothesis is supported by previous results obtained with conditional genetic experiments. Indeed, it was shown in conditional mutant mice lacking corticofugal axons that these axons are required for the guidance of thalamocortical axons across the PSPB (Chen et al., 2012). In a complementary study using conditional ablation of the embryonic thalamus, thalamocortical axons were shown to be necessary to guide corticothalamic axons into the corridor and toward the thalamus (Deck et al., 2013). However, the molecular mechanisms underlying this reciprocal interaction are not fully understood.

Importantly, we previously showed that draxin controls the guidance of thalamocortical axons by regulating the interaction of thalamocortical and corticofugal axons (Shinmyo et al., 2015). *Draxin* KO mice display severe defects in thalamocortical and corticofugal projections. Thalamocortical axons of *draxin* KO mice grow into the internal capsule, but the majority of them do not enter the cortex and instead either stall or turn laterally toward the external capsule. This phenotype is similar to that in conditional mutant mice lacking corticofugal axons, suggesting that draxin could mediate the interaction of thalamocortical and corticofugal axons. In *draxin* KO mice, corticofugal axons project normally toward the internal capsule at E14.5, while thalamocortical axons have already misrouted toward the external capsule at this stage (Figure 4). This result indicates that the pathfinding errors of thalamocortical axons precede those of corticofugal axons in *draxin* KO mice. *Draxin* is widely expressed in developing brains including in corticofugal neurons, while it is not expressed in thalamic neurons. The thalamocortical phenotype in *draxin* KO mice is rescued by the transgenic expression of *draxin* in the neocortex. These results suggest that draxin secreted from corticofugal axons is necessary for guidance of thalamocortical projections from the internal capsule to the neocortex.

Similar to the growth of neocortical axons, that of thalamic axons is promoted by low concentrations of draxin but inhibited by higher concentrations of draxin. The growth-promoting effect of draxin is absent in thalamic neurons from *Dcc* and *Neo1* double mutants. Consistently, *Dcc* and *Neo1* double mutants display a severe thalamocortical phenotype similar to that observed in *draxin* KO mice. Therefore, we have proposed that draxin from corticofugal axons promotes the growth of thalamocortical axons from the internal capsule to the neocortex and this effect of draxin is mediated through Dcc and Neo1 receptors (Figure 4; Shinmyo et al., 2015). It was shown that *netrin-1* is not expressed in neocortical neurons and that no obvious thalamocortical projection defects are observed in the internal capsule of *netrin-1* mutants (Braisted et al., 2000). Thus, draxin is likely to be a main player in Neo1- and Dcc-mediated guidance of thalamocortical projections into the neocortex. In our model, corticofugal axons are assumed to attract thalamocortical axons toward the PSPB. It is important to note that this model seems to be inconsistent with previous observations that neocortical and thalamic axons repel each other *in vitro* (Bagnard et al., 2001), and that bundles of thalamic and neocortical axons are segregated in the internal capsule and the intermediate zone. These facts have raised an intriguing possibility that thalamocortical axons might change their response to corticofugal axons from attraction to repulsion when they encounter each other in the internal capsule. This possibility might be explained by the concentration-dependent effects of draxin on thalamocortical axons. Further investigations are needed to clarify the molecular mechanisms governing reciprocal interactions between corticofugal and thalamocortical axons. It is interesting to note that commissural axons originated from the two hemispheres meet at the midline and continue to extend in opposite directions to reach their targets. This configuration seems to be similar to that of thalamocortical and corticofugal axons in the internal capsule. Since *draxin* is expressed in commissural neurons of the spinal cord and the neocortex, draxin might regulate axon-axon interactions of commissural neurons around the midline.

Thalamocortical axons originating from distinct nuclei in the dorsal thalamus project to the appropriate cortical areas through the internal capsule (Vanderhaeghen and Polleux, 2004; Garel and Lopez-Bendito, 2014). For instance, ventroanterior/ventrolateral axons project to the primary motor area, ventroposterior medial axons to the primary somatosensory area and dorsolateral geniculate nucleus axons project to the primary visual cortex. This topography is regulated by gradients of axon guidance cues including netrin-1, Slit1 and ephrin-A5 in the corridor and the striatum (Dufour et al., 2003; Bonnin et al., 2007; Powell et al., 2008; Bielle et al., 2011). *Netrin-1* mRNA is expressed in a rostral-high to caudal-low gradient in these structures (Bonnin et al., 2007; Powell et al., 2008). This graded expression of netrin-1 has been proposed to play a dual role in attracting rostral thalamic axons in a Dcc-dependent manner and repelling caudal thalamocortical axons in a Dcc-Unc5 receptor-dependent manner (Bonnin et al., 2007; Powell et al., 2008). *Draxin* mRNA is also expressed in the corridor and the striatum during thalamocortical development (Shinmyo et al., 2015), suggesting that draxin might interact with netrin-1 signaling to generate the topographic organization of thalamocortical projections.

## Glycoproteins and Draxin/Netrin-1 Signaling

We have proposed that draxin on the pial surface is critical for the guidance of commissural axons (Figure 2). Glycoproteins such as Dystroglycan and Heparan sulfate proteoglycans (HSPGs) may regulate the localization of draxin on the pial surface. Dystroglycan functions as a scaffold for extracellular matrix (ECM) proteins, which control distributions of secreted proteins (Wright et al., 2012; Lindenmaier et al., 2019). Indeed, ECM proteins that comprise the basement membrane show a fragmented and discontinuous pattern in a mutant of β-1,3-N-acetyl-glucosaminyltransferase-1 (B3gnt1), which is essential for glycosylation of Dystroglycan, suggesting that Dystroglycan is required for basement membrane integrity in the developing spinal cord. Furthermore, localization of Slit in the basement membrane and the FP requires the interaction between Dystroglycan and the C-terminal region of Slit (Wright et al., 2012). In the *B3gnt1* mutant, binding of alkaline phosphatase (AP)-tagged Slit2 to the basement membrane is absent. Thus, it is possible that Dystroglycan directly or indirectly regulates the distribution of draxin protein in the basement membrane of the spinal cord. HSPGs, components of the basement membrane, are also possible candidates to regulate the distribution of draxin protein. HSPGs were shown to interact with extracellular ligands such as those of the Wingless/Wnt and HH families and control their distributions (Lee and Chien, 2004; Masu, 2016; De Pasquale and Pavone, 2019). We previously reported that AP-tagged draxin binds many brain regions including the basement membrane in the cerebrum (Islam et al., 2009; Shinmyo et al., 2015). Addition of heparin drastically inhibited binding of draxin to the basement membrane (our unpublished data), suggesting that heparan sulfate (HS) mediates this binding. Therefore, it is plausible that glycoproteins could play crucial roles as regulators of the distribution of draxin on the basement membrane. Interestingly, draxin was shown to regulate basement membrane remodeling during epithelial-to-mesenchymal transition of cranical neural crest cells (Hutchins and Bronner, 2019). This finding might suggest that draxin itself regulates basement membrane integrity in the spinal cord and contributes to the localization of other axon guidance cues in the basement membrane.

It has also been shown that HSPGs play essential roles in modulating the action of axon-guidance ligands and receptors (Lee and Chien, 2004; Masu, 2016). There are several lines of evidence implicating HS and netrin-1/Dcc signaling. Heparin can bind both netrin-1 and Dcc (Serafini et al., 1994; Bennett et al., 1997). In addition, *in vitro* experiments suggested interaction of HS with Dcc is important for netrin-1 dependent commissural axon outgrowth (Keino-Masu et al., 1996; Bennett et al., 1997). Definitive evidence for a cell-autonomous role of HS in netrin-1-mediated axon guidance was demonstrated by using conditional knockout mice for *Ext1*, which encodes an enzyme essential for HS synthesis (Zak et al., 2002; Matsumoto et al., 2007). Ablation of *Ext1* in the dorsal spinal cord results in commissural axon pathfinding defects that share similarities with those of *netrin-1* and *Dcc* knockout mice. Consistently, *Ext1*-deficient dorsal spinal cord explants do not respond to netrin-1 *in vitro*. Furthermore, *Ext1*-deficient commissural neurons are incapable of inducing intracellular signaling downstream of netrin-1 and Dcc. Thus, expression of HS in commissural neurons is necessary for netrin-1/Dcc-mediated axon guidance.

Although the contribution of HS to draxin-dependent axon guidance is unclear, we suggest that HS might be involved in the concentration-dependent effects of draxin on cortical and thalamic axons (Shinmyo et al., 2015). Neurite outgrowth from cortical and thalamic neurons is promoted by draxin at low concentrations and inhibited at higher concentrations. The growth-promoting effect of draxin is absent in these neurons from *Neo1* and *Dcc* double-deficient mice, suggesting that this effect of draxin is mediated by Dcc and Neo1 receptors. Importantly, the inhibitory effect of draxin is not completely abolished in these neurons from *Neo1* and *Dcc* double-deficient mice, suggesting that, in addition to Dcc and Neo1, other receptors may be necessary for the inhibitory effect of draxin. Our preliminary data demonstrated that the inhibitory effect of draxin on cortical axons was absent following treatment with heparinase III, an enzyme that digests HS (Our unpublished data). This fact suggests that HSPGs might serve as low affinity receptors for draxin and participate in the conversion of draxin-induced attraction into repulsion.

## Intracellular Mechanisms of Draxin Signaling

It was shown that mice deficient for *MAP1B*, which encodes a microtubule-associated protein, have axon guidance defects in the corpus callosum and the hippocampal commissure (Meixner et al., 2000). These phenotypes are similar to those in *draxin* KO mice (Islam et al., 2009). Meli et al. (2015) demonstrated that draxin-induced growth cone collapse and inhibition of neurite outgrowth are suppressed in *MAP1B*-deficient cortical neurons. In addition, they showed draxin causes growth cone collapse through interaction with Dcc, inhibition of the phosphatidylinositol 3-kinase (PI3K)/Akt signaling pathway and activation of glycogen synthase kinase-3β (GSK-3β). Thus, it is likely that GSK-3β dependent MAP1B pathway is essential for repulsive draxin signaling. In contrast to draxin repulsion, molecular events in draxin attraction are still unclear. Further investigations are needed to elucidate the intracellular mechanisms underlying the bimodal effects of draxin.

## Draxin Signaling in Hippocampal Development

Our phenotypic analyses of *draxin* KO mice suggest that draxin has multiple roles in hippocampal development (Zhang et al., 2010; Tawarayama et al., 2018a, b, 2020). Importantly, loss of draxin leads to a reduction in the volume of the dentate gyrus, in addition to abnormal projections of the hippocampal commissure and mossy fibers. Consistently, enhanced apoptosis was observed in the developing dentate gyrus of *draxin* KO mice. Immunostaining with granule cell lineage markers revealed that the number of apoptotic neuroblasts was markedly increased in the dentate gyrus of *draxin* KO mice. It is known that Dcc belongs to the family of dependence receptors, which trigger apoptosis in the absence of ligands (Mehlen and Thibert, 2004). Indeed, Dcc is mainly expressed in neuroblasts of the dentate gyrus. Furthermore, our *in vitro* assays suggest that draxin suppresses Dcc-induced apoptosis in differentiating hippocampal neural cells. Thus, it is likely that draxin act as a dependent receptor ligand for Dcc to maintain and promote survival of neuroblasts in the dentate gyrus (Tawarayama, 2018; Tawarayama et al., 2018a).

## Conclusion and Perspectives

In this review, we discussed many possible roles of draxin and netrin-1 signaling in the development of neural circuits in the spinal cord and the brain. Further investigations are required to dissect these possibilities. In particular, spatially and temporally confined deletion of the *draxin* and/or *netrin-1* genes will be important to determine the precise roles of these molecules at multiple choice points for axon guidance in the developing CNS.

Studies of axon guidance signaling are critical to advance not only our knowledge of axon guidance mechanisms in the developing CNS, but also our understanding of human neurological disorders caused by axon guidance defects. Multiple human neurological disorders such as intellectual disability, autism spectrum disorders, and schizophrenia are known to be associated with developmental errors in axonal pathfinding (Nugent et al., 2012). It was suggested that mutations in the *NETRIN-1* or *DCC* gene cause congential mirror movements (CMM) because of inappropriate ipsilateral projections of the corticospinal tract from the hindbrain (Srour et al., 2010; Depienne et al., 2011; Meneret et al., 2017). CMM are involuntary symmetrical movements of one side of the body that mirror voluntary movements of the other side. Importantly, *draxin* KO mice are viable and fertile, even though they have drastic axon guidance defects in the brain. It will therefore be of great interest to investigate whether mutations in the *DRAXIN* gene are directly linked to human neurological disorders in future studies.

## Author Contributions

Both authors listed have made a substantial, direct, and intellectual contribution to the work, and approved it for publication.

## Conflict of Interest

The authors declare that the research was conducted in the absence of any commercial or financial relationships that could be construed as a potential conflict of interest.

## Publisher’s Note

All claims expressed in this article are solely those of the authors and do not necessarily represent those of their affiliated organizations, or those of the publisher, the editors and the reviewers. Any product that may be evaluated in this article, or claim that may be made by its manufacturer, is not guaranteed or endorsed by the publisher.
